# On the Superiority of Chopart's Operation and Excision of the Ankle in All Cases Admitting of Their Performance

**Published:** 1864-07

**Authors:** Henry Hancock

**Affiliations:** Surgeon to Charing-Cross Hospital.


### ORIGINAL ABSTRACTS AND SELECTIONS.

*On the Superiority of Chopart's Operation and Excision of the Ankle in all Cases Admitting of their Performance.* By HENRY HANCOCK, Esq., F. R. C. S, Surgeon to Charing-Cross Hospital.

It is not my intention to discuss the propriety of resecting joints, experience having long since settled this question in the affirmative; but I would inquire whether what has usually been called "conservative" surgery has progressed equally in the region of the foot and ankle-joint as in other parts of the body, and whether improvement has kept pace here with that which has obtained elsewhere.

Connected with this subject, there is no one in the United Kingdom to whom we are more indebted than to Mr. Syme, who, in the year 1843, first introduced to the profession his celebrated operation of exarticulation of the foot at the ankle-joint. Since that period, down to the end of the Crimean war, when Pirogoff promulgated his modification, Mr Syme's operation remained in the ascendant, as Chopart's valuable method was not supported, and the choice consequently reremained between amputation of the leg and Syme's operation, which he describes as follows:

"The incisions across the instep and foot should be curved, with the convexity forwards, and exactly opposite each other. A line drawn round the foot midway between the head of the fifth metatarsal bone and the melliolus externus will show their extent anteriorly, and they should meet a little way further back, opposite the malleolar projections of the tibia and fibula. Care should be taken to avoid cutting the posterior tibial artery before it divides into the plantar branches. The flaps thus formed are next separated from their subjacent connexions, which is easily effected except at the heel, where the firmness of texture occasions difficulty. The disarticulation being then readily completed, if the ankle-joint be sound, the malleolar processes should be removed by cutting pliers; but if the articular surfaces of the tibia and fibula be diseased, a thin slice of these bones should be sawn off. . . . . . The flaps are then brought together by means of sutures, preserving the pad of soft parts of the heel to form at the same time a covering for the end of the bones and a cushion whereon the patient could walk."

Great as was the acknowledged improvement of this operation over the old plan of amputation, it would seem to be open to objections. The irregularities of the protuberance of the os calcis, the intimacy with which the soft parts adhere thereto, and their comparative thinness at this point, render their detachment a matter of considerable difficulty, and they have not unfrequently been wounded in the attempt. Sloughing of the flap has followed in some instances, and among others in some of the earlier operations by Mr. Syme himself; but in the *Monthly Journal of Medical Science*, 1862, he states that he had performed the operation fifty times without this accident occurring.

To obviate these objections, M. Pirogoff, in the year 1852, introduced his modification, now known as "Pirogoff's operation." This differs from that of Syme, inasmuch as the posterior flap is not formed solely by soft parts, but consists of the posterior tuberosity of the os calcis with the insertion of the tendo-achillis. This flap is turned forwards, and the sawn surface of the os calcis brought in apposition with the cut surface of the tibia. The advantages claimed for this method are—that the tendo-achillis is not divided; that the soft parts not being detached from the tuberosity of the os calcis, the difficulties of this part of Syme's operation—the danger of wounding the flap and its subsequent sloughing—are thus avoided; that, from the posterior flap being, as it were, solid and not cupped, accumulations of pus are not likely to form in that situation; and lastly, that the leg is an inch and a half longer, from the posterior tuberosity of the os calcis left in the flap becoming united to the end of the tibia and fibula, thus lengthening the limb to that extent, and serving the patient as the point of support.

This operation, however, is liable to be followed by suppuration in the sheaths and sloughing of the divided tendons; and although this, to a certain extent, may be obviated by well-directed pressure, it is nevertheless a point of the greatest importance in governing our selection between the two operations. In February, 1858, Mr. Croft published in the *Lancet* the result of six cases of Pirogoff's operation—viz: four recoveries, two deaths; and it is well worthy of attention that whilst one patient died of granular disease of the kidney, the other died of secondary deposits of pus in various joints; and in all the four successful examples there was suppuration along the tendons of the leg.

There is still another and a very serious objection, however, which applies equally to both operations, and which should prevent our selecting them except in appropriate cases—viz: the necessary sacrifice of the foot and ankle-joint. But so great has been the prestige of these methods, so indiscriminately have they been employed, that we cannot read the hospital and other reports without feeling that the legitimate resources of surgery have not been made available to this region of the body, and I am sure that this feeling will not be confined to myself when it is learned that of forty-four cases submitted to Syme's and Pirogoff's operations which I have collected from various sources, in fifteen, or one third, the disease is stated to have been limited to the ankle-joint.

This has arisen partly from prejudice against Chopart's operation, and partly from the mistaken notion that excision of the ankle-joint is so difficult of performance and so hazardous that its adoption is scarcely safe or justifiable. The prejudices against Chopart's method, depending partly upon theoretical objection and partly upon error in the mode of performance, have prevailed, and a most valuable operation has consequently fallen into neglect. So, in like manner, resection of the knee, hips, elbow and shoulder-joints has frequently been performed; but we look in vain for resection of the ankle-joint, so completely have the minds of surgeons been engrossed by these imaginary fears, and so entirely have they ignored the value of the foot, or a portion of the foot, as a part of the animal economy.

The objection, however, made against Chopart's operation is, that the extensor muscles of the ankle having lost their opposing forces, and acting through the tendo-achillis, draw up the heel, and direct the cicatrix towards the ground, whereby the patient, obliged to bear his weight upon the tender cicatrix, is prevented walking by the agony induced, and suffers so much that he willingly undergoes secondary amputation. Mr. Syme, who performed secondary amputation at the ankle-joint in three cases, remarks: "You will observe that, as in all other cases of the same kind, anchylosis has taken place between the astragalus and calcis, whilst the latter has been previously drawn up by the action of the gastrocnemius, so as to prevent the patient from resting on the proper part of the stump."

In other cases, again, ulceration and exfoliation of the bone have occurred from tension of the flaps, necessitating the same untoward results.

Instances are also recorded in which, after this contraction had taken place, the tendo-achillis was divided, in the hope of remedying the mischief, but with so little success that Syme's operation was subsequently performed. Mr. Fergusson relates a case of this character.

Nevertheless, I still advocate Chopart's operation wherever the disease is located anteriorly to the os calcis and astragalus, and where an adequate flap can be obtained. I have now performed this operation four times, with the best results, and I attribute this success to the following mode of proceeding:

Making the upper flap at least an inch long, and carrying the under or plantar flap well on to the under surface of the toes, whereby, when there is much thickening, very nearly an additional inch is gained in that direction, and the junction of the flap is brought to the centre of the stump instead of the upper margin, to which the principal stress is referred. The flaps, moreover, being full and free, danger of sloughing is avoided; whilst, if required, they provide a sufficient covering for the whole or a portion of the navicular bone, which, when possible, should always be preserved, as by this means we not only obiviate the necessity of opening into the large synovial cavity of the astragalo-navicular joint, but we at the same time preserve the attachment of the calcaneo scaphoid ligament, and consequently the natural and firm support to the head of the astragalus, whilst we also make the stump more full and even. Again dividing the tendo-achillis at the time of operation, and not waiting to do so until contraction has already taken place, when such division is useless, as the parts have now become adherent and fixed, and it is too late to remedy the mischief.

The success, moreover, is greatly influenced by the situation at which the tendon is divided. When this is done near to the os calcis, where the fascia extending from the tendo-achillis on either side to the malleoli is dense and strong, and where the inability of the patient to rest his heel or foot on the ground during the antecedent disease has already induced a considerable amount of contraction, the consequent separation of the divided tendon is so slight that it quickly reunites, and the result is not to be relied upon. I always, therefore, select a point as near to the body of the muscle as practicable, where the fascia is less dense, and where the tendon is more under the influence of the muscular fibres?

Chopart's operation, performed in accordance with these suggestions, will prove as successful as any in surgery, and in support of this assertion I would refer to a case which made an excellent recovery, and left the hospital cured in two months.

I have frequently seen this patient since. He is now quite well, and able to walk and run up ladders as well (he says)

as ever he did. The plantar surface of the stump is perfectly horizontal, and the heel rests properly on the ground in standing or walking. Having had an artificial front of cork made to the stump, he wears a common boot, and sometimes walks several miles in the course of the day.

My other cases have been equally successful.

*Excision of the Ankle-Joint.*—I have now excised the ankle-joint in five cases; four times successfully, once unsuccessfully, the patient dying seven months after the operation of pthisis, induced by dissipation. My colleague, Mr. Bauvell, has also performed the operation. When we consider what has been done of late years in the surgery of other joints, when we also consider how much we may add to the comfort of, and what substantial benefits we may confer upon the patient by this operation, we may fairly inquire why a solitary exception is made in the case of the ankle-joint, and why the foot is so frequently and so unnecessarily sacrificed. Are the difficulties of this operation greater than those of Syme and Pirogoff, and is it more hazardous to the patient?

A very exaggerated notion would seem to exist upon this point. Even Mr. Fergusson, in his "Practical Surgery," says: "Under any circumstances I should consider such operations extremely difficult, and in most instances more dangerous to the patients than amputation at the ankle or leg." When such an opinion is expressed by so eminent a surgeon, it is not surprising that the operation should be regarded with disfavour; but I confess I do not understand in what this extreme difficulty or this great danger consists. When performed in the following manner, it is neither more difficult nor more dangerous than the generality of operations in surgery:

Commence the incision about two inches above and behind the external malleolus, and carry it across the instep to about two inches above and behind the internal malleolus. This incision is merely to divide the skin, and should not on any account penetrate beyond the fascia. Reflect this flap; next dislodge the peronei tendons from the groove at the back of the external malleolus, and cut through the external lateral ligaments of the joint, carrying the knife close to the edge of the bone. Having done this, cut through the fibula with the bone nippers, about an inch above the malleolus; remove this piece of bone by dividing the inferior tibio-fibular ligament, and then turn the leg with the foot on to its outer side. Now carefully dissect the tendons of the tibialis posticus and flexor communis digitorum from behind the internal malleolus, and, keeping the knife close around the edge of this process, detach the internal lateral ligament; then, grasping the heel with one hand and the front of the foot with the other, forcibly turn the sole of the foot outwards, by which the lower end of the tibia is dislocated and protruded through the wound. This done, cut off the end of the tibia with the common amputating saw, and then, with a small, thin metacarpal saw, introduced between the tendo-achillis and the upper articulating surface of the astragalus, remove the latter by cutting horizontally from behind forwards. Replace the parts *in situ*, close the wound carefully in front of the ankle, but leave the sides open to allow the discharge a free exit; apply water dressing, place the limb on a T splint, and the operation is completed.

There are no vessels divided in this operation, and, for obvious reasons, the greatest care should be taken not to wound either the anterior or the posterior tibial arteries.

I have already alluded to the risk attending Pirogoff's operation of subsequent suppuration in the sheaths of the divided tendons and sloughing of the latter, as exemplified by the six cases published by Mr. Croft. Regarding this point, Pirogoff says: "I fear nothing so much as this—viz: when the body of the muscle contracts and draws up the tendon, divided or half destroyed by suppuration, out of the sheath." Neither is Syme's operation free from this risk. In the two cases related by Butcher, one had a succession of secondary abscesses, the other had pyæmia, and was only saved by the skill and energy of this distinguished surgeon; whilst Mr. Fergusson remarks: "What with the violent inflammation extending up the leg, sloughing, secondary hemorrhage, death immediately dependent upon the operation, tardy healing of the wound, and defective condition of the stump, from languor of circulation and tenderness, so that it could not be pressed upon or made useful, I have formed a most unfavorable impression against it."

Excision of the ankle-joint is, for the most part, free from these defects. The nerves and arteries being preserved intact, sloughing does not occur; whilst the non-division of the tendons, and more especially the non-interference with the extensive sheath of the tendons in front of the joint, exempt the patient from the danger of suppuration in the sheaths and sloughing of the tendons, as well as from that danger of which Pirogoff seems to have so lively a dread.

In neither of my own cases has there been any sign of sloughing of the tendons or suppuration in their course, or of anything approaching to pyæmia. In one or two there was slight inflammation of the lymphatics, which subsided in a few days; and the amount of discharge has certainly not been more than, if so much as, what commonly follows excision of the knee-joint.

Cases in which the disease is situated mainly in the articulation, between the astragalus and os calcis, are not unfavorable for this operation. In my fourth case this complication existed. I removed the whole of the astragalus, behind its neck, and the diseased portion of the os calcis, and the child made a perfect recovery.

But if we meddle with the ankle-joint at all, never mind how limited the disease may be, we should always excise the entire joint, and not a portion merely; gouging can rarely succeed completely in removing the disease, and in addition inflicts an injurious amount of bruising.

In partial resections we expose our patients to the danger attending wounds of joints, whilst by complete excision the joint structure is got rid of, bone is brought in direct contact with bone, and the process of cure is rendered more simple and more certain. Besides total excision of the joint enables us to employ the saw, which should always be used where practicable, in preference to the gouge. We thereby obtain even surfaces, we bruise the parts less, and we are also enabled to form an opinion as to the soundness of the bones themselves.

Of the 5 patients upon whom I have operated for excision of the ankle-joint, 1 died seven months after the operation from pthisis; whilst of 33 cases collated from various sources I find that 21, or two-thirds recovered absoltutely, 7 suffered secondary amputation, and 5 died; but of these three are stated to have died of pthisis, one seven months, one three, and the third eight days after the operation. If the account of the two latter be correct, it might perhaps have been more judicious if the operation had not been performed at all. As regards the 7 secondary amputations, in 4 the joint was not entirely excised, but portions were allowed to remain.

Lastly, does excision of the ankle-joint afford as useful a limb to the working man as either Syme's or Pirogoff's operation? or does the removal of the malleoli and the subsequent destruction of the mortice-joint so weaken the part and so predispose it to secondary mischief as to render the foot an incumbrance rather than a benefit? In the first case upon which I operated the boy played at leap-frog within twelve months after the operation; there was very slight shortening of the limb—not more than a moderate addition to the sole of his boot could rectify. In the second, performed upon a man aged twenty-five, I frequently see him walking up the steep hill to Hampstead (a great strain even upon a sound ankle-joint) apparently with great ease. Whilst in the last case, which has just left the hospital, the boy walks about without difficulty. It is true that, as a matter of precaution, I have advised artificial support, in the shape of upright springs inserted on either side of the boot.

But what takes place in Pirogoff's operation occurs equally in this: there is either an ingrowing of bone into bone, of the tibia into the astragalus or calcis, or so dense a ligamentous union as at once to afford the requisite strength and a certain amount of motion in the part.

